# Caractéristiques de la population carcérale suivie en ambulatoire au service de psychiatrie du Centre Hospitalier National Universitaire de Fann, Sénégal

**DOI:** 10.11604/pamj.2021.39.221.23700

**Published:** 2021-07-29

**Authors:** Ibra Diagne, Véronique Petit, Khadim Seck, El Hadji Makhtar Ba, Ndèye Dialé Ndiaye-Ndongo, Aida Sylla, Mamadou Habib Thiam

**Affiliations:** 1 Service de Psychiatrie, Etablissement Public de Santé Mbour, Thiès, Sénégal,; 2 Institut de Recherche sur le Développement, Dakar, Sénégal,; 3 Centre National de Réinsertion Sociale Imam Assane Cissé, Kaolack, Sénégal,; 4 Service de psychiatrie, Centre Hospitalier National Universitaire de Fann, Dakar, Sénégal

**Keywords:** Détenus, population carcérale, troubles psychiatriques, Sénégal, Inmates, incarcerated subjects, psychiatric disorders, Senegal

## Abstract

**Introduction:**

les troubles psychiatriques affectent une grande proportion de la population carcérale. En effet, depuis plusieurs années, les conditions de détention dans les prisons sénégalaises sont décriées en évoquant notamment la surpopulation. Ces conditions jouent probablement un rôle déterminant dans la survenue des troubles psychiques dans cette population. Ce travail décrit les caractéristiques sociodémographiques et cliniques chez ces détenus suivis en ambulatoire au service de psychiatrie du Centre Hospitalier National Universitaire (CHNU) de Fann.

**Méthodes:**

l´étude était de type rétrospectif et descriptif. Les auteurs avaient colligé tous les détenus ayant consulté à l´Unité de la Consultation Externe du service de Psychiatrie du CHNU de Fann entre le 1^er^ janvier 2005 et le 31 décembre 2010, soit sur une période de six ans.

**Résultats:**

notre population d´étude était constituée de 62 détenus répartis en 92% d´hommes et 8% de femmes. L´âge moyen était de 32 ans, près de trois quarts des individus (72,6%) étaient célibataires. Leur situation professionnelle était précaire dans la majorité des cas et 69,3% d´entre eux n´avaient pas atteint le niveau d´étude secondaire. L´insomnie était le motif de consultation le plus important (29%) suivis des cas d´hallucinations acousticoverbales (22,6%). L´agressivité physique et/ou les menaces envers les codétenus étaient observées dans 17,7% des cas. Les principales catégories diagnostiques constatées étaient les troubles schizophréniques (32,3%) et les troubles dépressifs (27,4%). Entre 2005 et 2010, le nombre de consultations des détenus a été multiplié par trois/an passants de 7 à 19.

**Conclusion:**

il ressort de cette étude, que la population carcérale, suivie en ambulatoire en psychiatrie, est caractérisée par un âge jeune, majoritairement masculin et célibataire, de bas niveau d´instruction, de statut professionnel désavantagé. Un profil semblable a été dégagé dans la littérature internationale sur le même sujet. En l´état actuel du développement socio-économique et sanitaire du Sénégal, nous remarquons, que bien que le nombre de consultations des détenus augmente au fil des années, que seuls les individus avec des troubles mentaux sévères parviennent dans les services sanitaires spécialisés. Compte tenu de l´effectif de la population carcérale, des parcours biographiques des détenus, des conditions de détention et de l´absence de certaines pathologies, une enquête conduite dans les prisons s´avère nécessaire afin d´évaluer les besoins de soins de santé mentale des populations carcérales et les problématiques spécifiques qui pourraient les affecter.

## Abstract (en)

**Introduction:**

psychiatric disorders affect the highest number of incarcerated individuals. Indeed, detention conditions in Senegal have been criticized for several years by referring, in particular, to overcrowding. These conditions probably play a determining role in the occurrence of mental disorders in this population. This work describes the socio-demographic and clinical characteristics of these inmates followed on an outpatient basis in the Department of Psychiatry of the National University Hospital Center in Fann.

**Method:**

we conducted a retrospective and descriptive study. Data were collected from all the inmates presenting to the External Consultation Unit of the Department of Psychiatry of the National University Hospital Center of Fann between 1^st^ January 2005 and 31^st^ December 2010.

**Results:**

our study population consisted of 62 inmates, 92% men and 8% women. The average age of patients was 32 years, nearly three-quarters (72.6%) of individuals were single. In the majority of cases they were in a precarious employment situation and 69.3% of them had not completed secondary education. Insomnia was the leading reason for consultation (29%) followed by auditory verbal hallucinations (22.6%). Physical aggression and/or threats to fellow inmates were observed in 17.7% of cases. The main diagnostic categories found were schizophrenic disorders (32.3%) and depressive disorders (27.4%). Between 2005 and 2010, the number of consultations increased threefold/year, from 7 to 19.

**Conclusion:**

this study showed that the incarcerated population, followed on an outpatient basis in the Department of Psychiatry, were young, mostly male and single, with low level of education and disadvantaged professional status. Similar cases have been reported in the international literature. Considering Senegal’s socio-economic situation and health systems development, we note that, although the number of consultations has increased over the years, only inmates with severe mental disorders are followed in specialized health services. Given the number of incarcerated subjects, the biography background of these subjects, the conditions of detention and the absence of some diseases, a survey should be conducted in prisons in order to assess mental healthcare needs of incarcerated subjects and the specific issues that could affect them.

## Introduction

Depuis plusieurs années, les conditions de détention dans les prisons sénégalaises sont marquées par la vétusté des locaux, la surpopulation et la promiscuité. Les maisons d´arrêt et de correction (MAC) sont installées dans des bâtiments datant de l´époque coloniale, ces structures architecturales anciennes ne sont pas adaptées à leur fonction carcérale actuelle. Thioub *et al*. [1] décrivent en 1999 un système carcéral en crise faute de moyens et de personnels suffisamment nombreux et formés. Ces conditions ne constituent pas le cadre le plus propice au respect des droits des détenus. Outre des conditions de détention dégradées, la population carcérale souffre de difficultés d´accès à des conditions sanitaires satisfaisantes. En effet, il n´y a que deux médecins généralistes et un chirurgien-dentiste pour l´ensemble des prisons sénégalaises [2]. Ces facteurs principalement liés, contribuent à renforcer la vulnérabilité de cette population d´autant plus qu´elle est souvent oubliée dans les politiques sanitaires principalement en raison de son «invisibilité» sociale. La morbidité psychiatrique touche une grande proportion de la population carcérale [3, 4]. En effet la condition carcérale peut révéler, accroitre ou même déclencher une maladie mentale. L´Organisation Mondiale de la Santé (OMS) estime que près de 40% des prisonniers en Europe souffrent d´une forme de maladie mentale [5].

Dans les pays occidentaux, notamment en France, la prise en charge des détenus vivant avec un trouble mental est assurée par des services médicopsychologiques régionaux directement intégrés à la prison [6]. Ce sont pour la plupart des structures composées d´équipes pluridisciplinaires (médecins, psychiatres, infirmiers, psychologues, éducateurs spécialisés) [6]. Au Sénégal, malgré des conditions de détention difficiles, la santé générale des détenus n´est assurée que par des infirmiers. Cette catégorie de personnel, qui dépend de l´administration pénitentiaire, ne dispose ni des compétences, ni des moyens nécessaires afin de dispenser des soins spécialisés de qualité. En cas de suspicion d´une pathologie mentale chez un détenu, le régisseur de la prison met en place une escorte vers les structures psychiatriques afin que celui-ci puisse être soigné dans une structure adaptée. Dans le cadre de ce dispositif, le service de psychiatrie du Centre Hospitalier National Universitaire (CHNU) de Fann à Dakar (capitale du Sénégal) occupe une place importante.

## Méthodes

**Objectif général:** décrire les caractéristiques sociodémographiques et cliniques des troubles psychopathologiques retrouvés chez les détenus suivis en ambulatoire au service de Psychiatrie du CHNU Fann.

**Cadre de l´étude:** l´étude inclut la population des individus incarcérés au Sénégal et qui ont consulté dans le service de psychiatrie du CHNU de Fann de Dakar entre 1^er^ janvier 2005 au 31 décembre 2010 durant leur détention. C´est le plus ancien service de psychiatrie du Sénégal, il dispose d´une capacité d´accueil de 60 lits, avec une unité de pédopsychiatrie, d´addictologie et une unité de consultation. Ce service est situé dans le cadre d´un hôpital général de niveau III, qui sert de structure de référence pour de nombreuses formations sanitaires du pays dans un contexte où les ressources en psychiatrie sont insuffisantes et inégalement réparties sur le territoire sénégalais, la région de Dakar concentrant plus de 80% des ressources dans ce secteur [7, 8]. Sont accueillis dans ce service l´ensemble des cas de pathologie mentale quelle que soit leur origine et dont l´âge est supérieur à 16 ans. En outre, son implantation dans la capitale en fait une structure de soins pour les détenus des maisons d´arrêt et de correction (MAC) localisées dans la région de Dakar. Cette région concentre plus du tiers de la population carcérale et 8 maisons d´arrêt et de correction sur 36 y sont localisées [9].

**Type, période et contexte organisationnel de l´étude:** il s´agit d´une étude rétrospective, monocentrique et descriptive à partir d´informations extraites des dossiers médicaux de patients en situation d´incarcération au Sénégal entre 2005 et 2010. Les auteurs de cet article n´ont donc pas systématiquement participé à la collecte des informations mobilisées dans ce travail, bien que certains en raison de leur appartenance au système de santé psychiatrique sénégalais aient déjà établi des observations cliniques et des diagnostics concernant des individus appartenant à la sous-population observée.

**Population d´étude et critères d´éligibilité:** l´étude concerne l´ensemble des patients détenus sur le territoire du Sénégal qui ont été reçus au service de psychiatrie du CHNU de Fann pour des soins ambulatoires. Nous avons inclus dans l´étude les patients détenus ayant consulté au cours d´une période de 6 ans allant du 1^er^ janvier 2005 au 31 décembre 2010. Après l´examen des dossiers médicaux, il a été décidé d´exclure de cette étude les patients dont le dossier médical était incomplet ou lorsque le dossier n´était pas disponible dans les archives de l´unité.

**Variables:** une fois les dossiers médicaux complets sélectionnés, un interne en psychiatrie formé à cet effet a opéré la saisie informatique des variables sociodémographiques à partir d´une fiche de recueil de donnée standardisée. Celle-ci incluait les variables suivantes: l´âge, le sexe, le statut matrimonial, le niveau d´instruction, la profession avant l´incarcération; des variables cliniques (motifs de consultation); des variables diagnostiques ainsi que le nombre de consultations par année. Pour les diagnostics, le chapitre F (troubles mentaux et troubles du comportement) de la 10^e^ révision de la Classification Internationale des Maladies (CIM-10) de l´Organisation mondiale de la santé a été notre référence diagnostique.

**Analyse statistique:** l´objectif de cette étude a été de déterminer le profil des patients détenus suivis à titre externe au Service de Psychiatrie de Fann. Pour chaque variable, les données recueillies ont été codées afin de préserver l´anonymat des patients conformément aux règles déontologiques. Elles ont été ensuite saisies et traitées à partir du logiciel de traitement Sphinx2+. Les figures et les tableaux ont été réalisés avec le logiciel Excel.

**Biais:** cette étude repose sur une analyse secondaire de données. Les auteurs ne sont donc pas en mesure de contrôler les biais dépendants des conditions de déclaration au moment de la création du dossier médical. Néanmoins les patients sont des détenus et leur identité (sexe, âge, statut matrimonial, niveau d´éducation, profession) a été contrôlée par les administrations judiciaires et pénitentiaires. On peut donc estimer que les variables sociodémographiques ont été déclarées et vérifiées avec justesse.

Les examens cliniques et les diagnostics ont été établis par des médecins psychiatres expérimentés dans la structure de référence du Sénégal, établissement qui dispose d´une réputation internationale et contribue à la formation de psychiatres de toute la sous-région (Afrique de l´Ouest et centrale). On peut émettre une réserve par rapport au fait que le diagnostic ait été posé par un seul clinicien et non pas de manière collégiale ce qui aurait permis de discuter les observations cliniques entre praticiens.

Il faut également noter qu´en raison du statut particulier des patients, ceux-ci n´étaient pas soutenus psychologiquement par un accompagnant qui est fréquemment en temps ordinaire un membre du cercle familial proche. Une présence policière était également requise afin d´éviter toute tentative d´évasion dans le couloir ou dans le bureau de consultation. Néanmoins, les patients étaient reçus avec toute l´attention requise par le personnel soignant, la consultation constituait alors un espace privilégié d´expression et de reconnaissance.

Outre une meilleure connaissance de la santé psychique de la population carcérale au Sénégal, cette étude permet de valoriser des données sanitaires disponibles alors que le système statistique national se caractérise par un déficit d´indicateurs en santé mentale à l´échelle nationale. Les soins en santé mentale ne relèvent pas en effet de la santé primaire, mais de services spécialisés, par conséquent les consultations en psychiatrie ne sont pas enregistrées dans le système d´information sanitaire du Ministère de Santé (DHIS-2) ce qui contribue à une forme d´invisibilité de la santé psychique. A ce jour il n´existe pas de statistiques ou d´enquêtes permettant de décrire la santé mentale de la population du Sénégal. Des recherches ponctuelles sont disponibles, mais elles ne concernent pas la population carcérale dont la santé reste largement méconnue.

## Résultats

Dans le champ couvert par l´étude, soixante-deux dossiers de détenus ont été retenus en raison de leur complétude dans la période indiquée. La moyenne d´âge était de 32 ans (âge médian = 31,5 ans) avec des extrêmes allant de 16 à 65 ans. La tranche d´âge 25-35 ans était la plus représentée (46,7%). Le sexe masculin était majoritaire (92%) avec un sexe ratio de hommes sur femmes à 11,4 (57 hommes pour 5 femmes). Par rapport au statut matrimonial, les célibataires représentaient 72,6% des cas. Plus de la moitié de la population d´étude se caractérise par un faible niveau d´étude. En effet, 69,3% des patients n´avaient pas accédé à un enseignement secondaire. Au moment de leur incarcération ces patients étaient peu qualifiés, car 46,8% d´entre eux étaient sans profession et 38,7% étaient ouvriers journaliers (Tableau 1).

**Tableau 1:** caractéristiques sociodémographiques des détenus suivis à la consultation externe de psychiatrie du CHNU de Fann du 1^er^ janvier 2005 et le 31 décembre 2010 (N=62)

	Effectifs (n)	Proportions (%)
**Sexe**		
Masculin	57	92
Féminin	5	8
**Tranches d'âge**		
[16 – 25 ans]	8	12,9
[26 – 35 ans]	29	46,7
[36 – 45 ans]	12	19,4
[46 – 55 ans]	7	11,3
[56 – 65 ans]	6	9,7
**Situation matrimoniale**		
Célibataires	45	72,6
Marié (es)	11	17,7
Divorcé (es)	4	6,5
Veufs (ves)	2	3,2
**Profession avant incarcération**		
Sans professions	29	46,8
Ouvriers journaliers	24	38,7
Travail libéral	8	12,9
Fonctionnaires	1	1,6
**Niveau d'étude**		
Analphabète	24	38,7
Primaire	19	30,6
Secondaire	14	22,6
Supérieur	5	8,1

Sur le plan clinique, l´insomnie était le motif de consultation le plus fréquent (29%). Les états d´agitation psychomotrice à type d´agressivité physique et de menaces envers les codétenus ont été recueillis dans 17,7% des cas. Les hallucinations acousticoverbales ou les comportements hallucinatoires constituaient 22,6% des cas. Les tentatives de suicides et les idéations suicidaires représentaient 8,1% des observations cliniques (Tableau 2).

**Tableau 2:** répartition des détenus de l’étude suivis du 1^er^ janvier 2005 et le 31 décembre 2010 en fonction des motifs de consultation

Motifs de consultation	Effectif (n)	Proportion (%)
Insomnie	18	29,0
Agitation psychomotrice (agressivité)	11	17,7
Troubles du comportement	9	14,5
Hallucinations	14	22,6
Angoisse	5	8,1
Idées suicidaires	4	6,5
Tentatives de suicide	1	1,6

**Figure 1:**
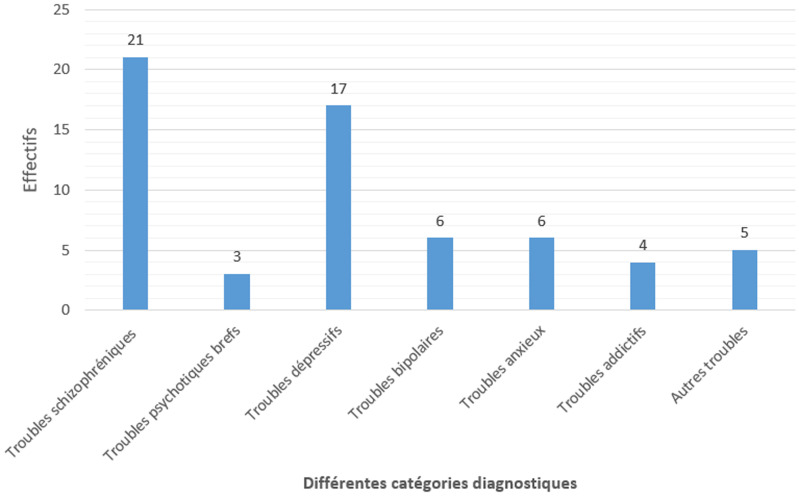
distribution des détenus de l’étude par catégorie diagnostique retenue

Par rapport aux pathologies mentales retenues, les troubles schizophréniques prédominaient avec 33,9%, ils étaient suivis par les troubles dépressifs (27,4%). Les troubles psychotiques brefs, les troubles bipolaires et les troubles névrotiques avaient une représentativité inférieure à 10% pour chacun d´eux (Figure 1).

**Figure 2:**
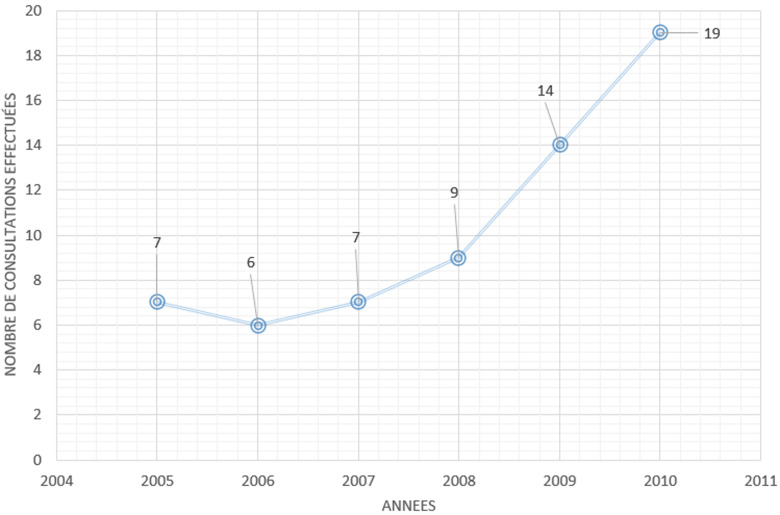
évolution du nombre de consultations des détenus suivie entre le 1^er^ janvier 2005 et le 31 décembre 2010 selon les années

Concernant l´évolution du nombre de consultation des détenus, elle avait augmenté au fur et à mesure des années. Cette évolution était globalement régulière de 2005 à 2010 avec un taux majoré dans les dernières années de la période de l´étude. Le nombre de consultations a été multiplié par trois, passant de neuf consultations en 2008 à 19 en 2010 (Figure 2).

## Discussion

Durant la période de l´étude, soixante-deux dossiers de détenus ont été retenus. Alors que la population carcérale à un moment précis de l´année s´élève en 2011 à presque 20800 individus [9], force est de constater l´extrême rareté des consultations psychiatriques à l´époque de l´étude.

Le sexe masculin représentait 92% de la population d´étude. Cette prédominance des hommes pourrait s´expliquer par les caractéristiques de la population carcérale sénégalaise qui est majoritairement masculine soit 96,5% [2]. Le sexe est apparemment la variable sociodémographique la plus citée dans la littérature médicale en matière d´emprisonnement [10, 11]. Houssani *et al*. dans leur étude sur les caractéristiques sociodémographiques de 93 détenus suivis à la consultation externe de psychiatrie à l´hôpital Raki relevaient que le sexe masculin représentait 95,7% des cas [12]. Une autre étude faite par Nanema *et al*. sur les aspects épidémiologiques et cliniques des troubles psychiatriques en milieu carcéral au Burkina Faso avait montré que les hommes détenus représentaient 95% de la population carcérale [13]. Ces résultats se superposent et nous interpellent, ils nous questionnent afin de savoir si les hommes sont plus exposés aux risques de développer des conduites agressives et des troubles du comportement. Pour l´instant peu de recherches africaines se sont penchées sur cette question. Dans certains ouvrages internationaux, comme celui de Brousolle [14] sur la délinquance et la déviance, l´auteur parlait de «chromosome du crime». Cependant avant d´évoquer des facteurs génétiques particulièrement déterministes, il semble important d´évoquer des facteurs socioculturels relatifs à la place des femmes dans la société sénégalaise et à leur rôle protecteur d´épouse et de mère, alors que les hommes doivent assumer le rôle traditionnel de pourvoyeurs des ressources des ménages. D´autre part, les détenus se caractérisent par une origine sociale modeste (niveau d´instruction, profession) qui peut induire des conditions de vie précaires exposant à une certaine vulnérabilité.

L´âge des patients détenus de notre population d´étude était compris entre 16 à 65 ans avec une moyenne d´âge de 32 ans. Ce résultat est similaire à celui de Faure et Prieto [15]. Dans cette étude réalisée en France dans les services médicopsychologiques régionaux, les auteurs relevaient une moyenne d´âge de 31 ans. Notre population de détenus était composée d´adultes jeunes. Cette jeunesse pourrait s´expliquer par les caractéristiques de la population sénégalaise en générale dont l´âge médian en 2017 était de 17,8 ans [16]. Plusieurs études [17, 18] avancent que les adultes jeunes sont plus violents que le reste de la population, ceci concerne également les malades mentaux. Certains auteurs stipulaient que le risque de passage à l´acte était plus élevé chez les patients âgés de 30 à 40 ans [17, 18].

Près de trois quarts de notre population d´étude étaient constitués par des célibataires avec un taux de 72,6%, ce qui est cohérent avec sa jeunesse. En effet, le statut de célibataire est considéré par plusieurs auteurs comme un facteur de risque de passage à l´acte violent comme en témoigne Klassen et O’Commor [19], qui avancent que le célibat représente un risque élevé de violence en comparaison avec les sujets vivants en couple. Devant ces constats, l´hypothèse du rôle stabilisateur social du mariage prend tout son sens. Toutefois, la question reste complexe car les données existantes ne permettent pas de dire s´il s´agit du mariage ou du simple fait d´être en couple même sans passer par l´institution sociale que constitue le mariage. De toute façon, les liens relationnels forts semblent jouer un rôle protecteur dans la survenue d´acte de violence.

La situation professionnelle avant l´incarcération était précaire dans la grande majorité des cas, rappelons en effet que 46,8% des détenus étaient sans profession et 38,7% des ouvriers journaliers. Ces résultats sont superposables à l´étude de Houssani *et al*. sur le même sujet [12]. Une autre étude menée auprès des Services Médicopsychologiques Régionaux en France avait montré que la moitié des détenus n´avait pas de profession à leur entrée en détention [6]. L´absence d´emploi salarié ou d´insertion dans le secteur informel qui caractérise l´économie sénégalaise de ces jeunes hommes implique l´absence de ressources financières et donc de reconnaissance sociale ce qui les place en difficulté vis-à-vis de leur famille et de leur communauté. Si ces jeunes ont déjà effectué plusieurs peines de prison, les résultats observés peuvent s´expliquer par les peurs de la société face à cette catégorie de la population, craintes qui rendent difficile toute réinsertion socioprofessionnelle. En criminologie, la situation professionnelle est un paramètre d´une grande importance. Ainsi certains auteurs considèrent que l´inadaptation et l´instabilité au travail sont des facteurs prédicteurs criminologiques de l´infraction pénale [20, 21].

Le niveau d´étude était peu élevé puisque dans la majorité des cas (69,3%) les patients n´avaient pas été au-delà du niveau primaire. Plusieurs auteurs relèvent également cet état de fait [4, 6, 12]. Ainsi une étude faite en France en 2007 sur la trajectoire scolaire des détenus avait montré que près de la moitié des prisonniers étaient sans diplôme, uniquement 3% d´entre eux détenaient un diplôme universitaire [22]. Ce constat de parcours scolaires décousus et chaotiques des détenus s´expliquerait par l´appartenance à un milieu socio-familial désavantagé. En outre, le niveau d´étude bas chez les détenus au Sénégal détermine en partie une situation professionnelle précaire.

Sur le plan clinique, l´insomnie était au premier rang des motifs de consultations des détenus avec un taux de 29%. Ce résultat est comparable à celui trouvé par Manzenera et Senon [23], dans une étude sur la psychiatrie de liaison en milieu pénitentiaire, où l´insomnie représentait 27% des cas. Cependant, Nanema *et al*. [13] avaient enregistré une prévalence de 51% de l´insomnie chez les détenus en milieu carcéral à Ouagadougou au Burkina Faso. Le fait que notre étude se déroule dans une unité de psychiatrie générale invite à être prudents dans les comparaisons avec les données issues de structures pénitentiaires. Néanmoins la fréquence de l´insomnie chez les détenus est une donnée classique [23, 24]. La rupture des relations sociales, les pensées réflexives sur les actes commis ou l´attente anxiogène de jugement ont été considérées comme des facteurs favorisants l´insomnie chez les détenus [4]. En outre, ce symptôme conduit parfois à de véritables attaques de panique avec un risque de raptus anxieux ou de passage à l´acte auto et/ou hétéro-agressif.

Au deuxième et troisième rang des motifs de consultation, apparaissent successivement des cas d´hallucination ou de comportements hallucinatoires (22,6%) et des états d´agitation psychomotrice à type d´agressivité physique et de menace envers les codétenus (17,7%). Une des problématiques les plus citées dans la littérature chez les détenus est la violence. Une étude effectuée dans une prison de femmes aux États-Unis en 2014 montrait qu´un cinquième des détenues (20,5%) était concerné par des actes de violence physique entre détenues [24]. Cette même étude stipulait que les personnes souffrant de pathologie mentale étaient plus fréquemment impliquées dans ces incidents violents [24]. Ce constat expliquerait en partie la fréquence de l´agressivité physique comme motif de consultation en psychiatrie. Ces observations posent avec acuité la problématique de la légitimité de leur présence en institution carcérale. Des expertises médicales ne seraient-elles pas nécessaires afin de transférer ces personnes en détresse psychique en institution psychiatrique? La question reste entière et nécessite des dispositions particulières en commençant par une évaluation psychologique initiale et une expertise psychiatrique dès l´incarcération, voire à intervalles réguliers afin de mesurer les effets psychiques de la condition de prisonnier.

Outre la morbidité, la question de la mortalité se pose à propos des tentatives de suicide, qui représentent 1,6% des cas. Ici nos résultats divergent avec ceux observés dans les pays développés où les tentatives de suicide en détention posent un problème majeur. Une étude réalisée en France en 2002 sur les soins pénalement obligés [13] avait révélé une proportion importante de tentatives de suicide (37,7%) et où le suicide représentait 92% des causes de décès en prison. Cet écart trouve son explication dans plusieurs facteurs socioculturels. D´une part, le suicide constitue encore comme un phénomène tabou et réprimé dans nombre de sociétés africaines où la vie est considérée comme sacrée. Dans un contexte de forte religiosité, mettre fin à sa vie est tributaire d´une sanction divine. D´autre part, malgré l´émergence de conduites de plus en plus individualistes [25, 26], les solidarités familiales et l´entraide constituent une protection pour l´individu en situation de privation de liberté.

Du point de vue du profil diagnostic, on remarque la relative rareté des troubles psychiatriques mineurs, moins de 10% pour les troubles psychotiques brefs, troubles bipolaires, troubles anxieux et les troubles addictifs. Les troubles schizophréniques (32,3%) prédominent chez la population d´étude, viennent ensuite les troubles dépressifs (27,4%). La prévalence élevée des troubles schizophréniques chez les détenus observés est aussi relevée dans plusieurs régions du monde [13, 27-29]. Plusieurs facteurs expliquent cette fréquence dont le plus fréquemment cité dans la littérature est le contexte d´emprisonnement lui-même [30]. De plus, comme les troubles schizophréniques sont des pathologies fréquemment bruyantes et associées à des agressions à l´encontre d´autrui, elles sont mal tolérées par l´entourage, d´où le recours aux soins spécialisés au cours de l´évolution de la maladie.

En dehors des troubles schizophréniques, les troubles dépressifs (27,4%) occupaient le second rang des diagnostics les plus retenus chez les détenus. Nos résultats sont comparables à ceux retrouvés dans la littérature. Molink [28] avait retrouvé un taux de dépression de 25,8% chez des détenus au Canada. Un taux de 40% de dépression dans une étude menée sur la prise en charge des détenus au centre hospitalier universitaire de Montperrin [30] a été relevé. La variation des taux de dépression notés dans les différentes études s´expliquerait par le mode de recrutement des détenus. Néanmoins ces taux sont élevés dans toutes les études.

**Limites:** les résultats collectés et présentés dans notre travail ont été obtenus à partir des informations disponibles dans les dossiers médicaux des patients. Ces derniers n´étaient pas toujours complétés de manière exhaustive et précise, de plus le diagnostic chez les patients n´était pas toujours posé de manière collégiale. A cette première limite, s´ajoute l´effectif réduit des patients colligés dans ce service psychiatrique durant cinq années. Cet état de fait peut s´expliquer par différents facteurs: un suivi médical général insuffisant des détenus faute de ressources humaines compétentes, un manque d´attention aux questions de santé psychique dans les prisons engendrant l´absence de diagnostics de certaines pathologies mentales, et donc une moindre prise en charge des troubles psychiques. Cependant, malgré les limites que nous venons de rappeler, l´apport d´une telle étude préliminaire est d´un intérêt capital pour la connaissance du profil des patients détenus qui recourent au système de soins et pour évaluer les manques dans la prise en charge psychiatrique des détenus.

## Conclusion

La population de cette étude se caractérise par un profil jeune, majoritairement masculin et célibataire, un bas niveau d´instruction et un statut professionnel désavantagé. Ce profil est semblable à celui qui émerge de la revue de la littérature internationale. Sur le plan clinique, on remarque que seuls les détenus ayant des troubles mentaux sévères, c'est-à-dire des troubles schizophréniques et dépressifs, arrivent en consultation dans les formations sanitaires. Ces résultats mettent clairement en évidence des besoins de soins plus élevés que ceux qui illustrent la demande actuelle de prise en charge. Cette sous-estimation des besoins en santé mentale dans les institutions carcérales pose la question de l´équité en matière d´accès aux soins de santé au sein de la population du Sénégal et des moyens consacrés à la condition carcérale. La place de la santé mentale concerne par ailleurs autant les détenus que le personnel intervenant dans les MAC celui-ci étant confronté quotidiennement aux différentes formes de violences liées au contexte dégradé de l´enfermement au Sénégal. Une politique carcérale digne de ce nom dans un Etat respectueux des droits humains ne peut faire l´impasse sur cette question.

### Etat des connaissances sur le sujet

- La surpopulation dans les prisons africaines en général et spécifiquement au Sénégal;
- La morbidité psychiatrique touche une grande proportion de la population carcérale.

### Contribution de notre étude à la connaissance

- La faible proportion des détenus qui sont suivis en consultation psychiatrique hospitalière;
- Les troubles mentaux sévères chez les détenus sont les plus fréquemment trouvés en milieu psychiatrique;
- La nécessité d´effectuer des investigations en milieu carcéral afin de mesurer l´ampleur des troubles psychiatriques et d´apporter des réponses dès le début de l´incarcération.
